# Real-Time Diffusion of Information on Twitter and the Financial Markets

**DOI:** 10.1371/journal.pone.0159226

**Published:** 2016-08-09

**Authors:** Ali Tafti, Ryan Zotti, Wolfgang Jank

**Affiliations:** 1 Department of Information and Decision Sciences, College of Business Administration, University of Illinois at Chicago, Chicago, Illinois, United States of America; 2 Capital One Corporation, Richmond, Virginia, United States of America; 3 Department of Information Systems Decision Sciences, Muma College of Business, University of South Florida, Tampa, Florida, United States of America; Universidad de las Palmas de Gran Canaria, SPAIN

## Abstract

Do spikes in Twitter chatter about a firm precede unusual stock market trading activity for that firm? If so, Twitter activity may provide useful information about impending financial market activity in real-time. We study the real-time relationship between chatter on Twitter and the stock trading volume of 96 firms listed on the Nasdaq 100, during 193 days of trading in the period from May 21, 2012 to September 18, 2013. We identify observations featuring firm-specific spikes in Twitter activity, and randomly assign each observation to a ten-minute increment matching on the firm and a number of repeating time indicators. We examine the extent that unusual levels of chatter on Twitter about a firm portend an oncoming surge of trading of its stock within the hour, over and above what would normally be expected for the stock for that time of day and day of week. We also compare the findings from our explanatory model to the predictive power of Tweets. Although we find a compelling and potentially informative real-time relationship between Twitter activity and trading volume, our forecasting exercise highlights how difficult it can be to make use of this information for monetary gain.

## Introduction

Financial firms and academic researchers have recently begun to study the predictive value of information gathered from social media [1–3]. A recent paper [3] provides an excellent review of related research. The study and tracking of chatter on large-scale social networks such as Twitter has spawned new industries that aim to harness valuable information about popular and consumer sentiment [4]. Social networks also provide channels for widespread chatter and speculation about the financial viability and success of firms on the stock market.

Although researchers have begun to study the relationship between patterns observed on Twitter and stock market activity at daily levels of aggregation, we do not yet have much understanding of *intra-day responses* of the stock market in relation to the spread of news on Twitter. Such effects may be transitory and dissipate within hours or minutes, and it is precisely these effects that many algorithmic traders would like to exploit in real time. Moreover, interesting details about the rates at which signals propagate and dissipate on the Twitter social network and in the financial markets can be observed at time-resolutions of minutes; these may be missed entirely when the data is aggregated at the daily level.

In this study, we consider the real-time relationship between chatter on Twitter and the trading volume of 96 firms listed in the Nasdaq 100, during 193 days of trading in the period from May 21, 2012 to September 18, 2013. In contrast to research in [1], in which predictions are done at a daily level for the entire Dow Jones Industrial average, our study is at the firm level. Further, we consider a more precise intra-day granularity of observations at the level of ten-minute periods for each firm during trading hours, resulting in a panel time-series data set of 618,261 observations. Over all of these 618,261 observations, we counted a total of approximately 35 million Twitter messages mentioning the 96 firms. Unlike research findings presented in [1], we do not consider the sentiment within the messages. Thus, in the scope of the current study, we take trading volume as the dependent variable of interest, which can be sensitive to the *amount* of social media chatter about a firm even if we are agnostic about the qualitative features of the sentiment expressed in the chatter. Our analysis differs from common panel data analyses in that we are interested in spikes representing unusual Twitter activity. In fact, while most analyses in prior information systems (IS) literature focus on the average behavior (“regression to the mean”), we are particularly interested in events that are unusual and that would be considered a statistical outlier by most standards. That is, in this study we are interested in events that lead to unusually high levels of Twitter activity. We investigate whether such Twitter spike events lead to sudden spikes in trading volumes, a common measure of market activity levels, in the stock for each firm.

Introductory textbooks of statistics often conclude that outliers might be discarded from analysis or investigated separately from the remainder of the data [5]. The reason is that outliers often unduly influence the regression model and hence skew the results [6]. In this work, we take on a different point of view. We consider outliers and unusual events our main focus of interest. In that sense, one of the contributions of this work is the suitable identification of events that are unusual in nature.

The detection and analysis of unusual events is not quite as common as the investigation of the mean behavior, and its discussion has gained more attention outside the IS literature, e.g. in biosurveillance [7]. In fact, much of the work on outlier detection can also be found in the context of anomaly detection [8], and it often focuses on areas such as financial fraud, network intrusion, or faults in manufacturing [9]. Our focus is on social media. We emphasize that the ultimate objective of this paper is not to merely identify unusual events; rather, our goal is to identify such events and consequently examine their association with oncoming trading activity in the stock market within the subsequent hour (i.e. the subsequent 10 to 50 minutes); a period of time in which actionable decisions may be possible. To that end, one of our main challenges is the creation of a suitable test bed.

Unusual Twitter events (i.e. events with an unusually high number of Tweets) are rather rare; most of the time, Tweets mentioning specific firms arrive at a moderate rate. We illustrate this with two mini-case examples below; but the general pattern of low Twitter mentions of a firm punctuated by well-defined and clearly visible spikes is one we see in virtually every full time-series of Twitter activity for firms in our sample. In order to properly compare the effect of unusual Twitter events with that of the “typical” rate of Tweets, it is necessary to create suitable counterfactuals. In this work, we adopt a quasi-experimental design, in which we identify observations featuring firm-specific spikes in Twitter activity, and randomly assign each observation to a ten-minute increment matching on the firm symbol and a number of repeating time indicators that match spikes to non-spikes to within the same day and time of week at the half hour level. We examine the extent that unusual levels of chatter on Twitter about a firm portend an oncoming surge of trading of its stock within the hour, over and above what would normally be expected for the stock in a given day of week or time of day.

Our results suggest that, through monitoring of chatter on Twitter about firms listed on the Nasdaq 100, observing spikes of chatter affords a reliable and non-trivial amount of foresight into oncoming surges in trading volume. Sometimes Twitter messages have causal impacts. A recent hoax that spread on Twitter claimed that President Obama was injured by an explosion at the White House; this caused a temporary drop of 150 points in the Dow Jones industrial average [4]. Generally, however, we do not posit that messages on Twitter have causal impacts on the stock market; but rather that the monitoring of chatter on Twitter can be potentially useful for modest improvements in real-time predictions of oncoming surges in trading activity. Anomalies of chatter on Twitter can also reveal certain competitive dynamics within industries; for example, when product announcements of one firm impact their suppliers or rivals, as we discuss below with specific examples related to Garmin and Akamai in June 2012.

This study has two primary objectives as a research contribution. First, the study represents a microscope upon the diffusion of information in social networks that becomes observable at a resolution of minutes. Second, our approach allows a better understanding of how social networks and financial markets simultaneously respond in real-time to external events, drawing contrasts in the speed in which information is propagated in both types of spaces. Unlike traditional time-series approaches that consider spikes as anomalies in the data that need to be removed, we treat spikes as central to the analysis because they represent real reactions to news and have tangible impacts that should not be ignored. We ask the following research questions: 1) To what extent is there a predictive relationship between the spread of new information on Twitter about a firm, and the reaction in the financial markets? 2) Is there a measurable and predictable difference in the time it takes for new information to spread in Twitter and the time it takes for that information to be absorbed in the financial markets? We also compare the findings from our explanatory model to the predictive power of Tweets in attempt to address some of the ongoing conundrums in the IS literature [10].

## Data

We study the real-time relationship between chatter on Twitter and the trading volume of Nasdaq 100 firms during 193 days of trading in the period from May 21, 2012 to September 18, 2013. Data was not collected during weekends and holidays, when financial markets were closed. One author operated the data-collection program on a daily basis; but data collection was sometimes disrupted during extended travel or disruptions in internet connectivity. Since our emphasis is on investor rather than consumer sentiment, we excluded the most common household names listed in the Nasdaq 100 from this study: Facebook, Microsoft, Intel, Google and Apple. We used the Nasdaq 100 list as of May 1, 2012 (Facebook had not yet been listed). Among Nasdaq 100 firms, these particular firms have a dominant presence on Twitter in terms of their frequency of being mentioned. The comments we observed on Twitter mentioning these household names are seldom directly related to their financial performance and more often represent consumer sentiment.

During the 193 trading days, we collected a stream of continuous Twitter feeds of messages that mention the common names of 96 firms listed in the Nasdaq 100 index (for example, “Expedia” for “Expedia, Inc.”, “Lam Research” for “Lam Research Corporation”). We counted the number of Twitter messages in which each firm is mentioned in each ten-minute period. We use the open third-party application-programming interface Twitter4J to connect to Twitter’s public “garden hose”, a random 1% stream. In total, we counted 35 million messages mentioning the 96 firms during the study period. Using an automated screen scraper, we also gathered Yahoo! Finance data at the beginning and end of each ten-minute period, in particular for the price and trading volume of each stock. Our data collection methods comply with the terms of service of both Twitter and Yahoo.

We restricted the sample to the hours of trading from 10 am to 4 pm eastern standard time (EST), eliminating observations during the first half hour of trading when daily trading volumes are highly irregular. Each unit of observation represents a ten-minute period during all but the first half-hour in each day of trading, for each of the 96 firms over the 193 trading days. This results in a total of 618,261 observations that we draw from for this study.

Adopting a quasi-experimental design, we identified observations featuring firm-specific spikes in Twitter activity (which we call henceforth a Twitter spike), and assigned each Twitter spike to a randomly selected ten-minute increment matching on the firm and a number of repeating time indicators. This resulted in a final data sample of 11,241 observations in 5,480 treatment-control group pairings for 94 firms (two out of 96 firms did not have sufficient Twitter activity to form such pairings). Table 1 shows an example of a treatment-control group pairing taken directly from our final data sample. On February 14, 2013, our procedure determines the 80^th^, 90^th^, and 99^th^ percentile levels for the firm Adobe to be 0.185, 0.248 and and 0.802 Twitter mentions per second (TPS), respectively. As we elaborate below, percentile calculations are calculated with reference to an expanding training-period window that ends prior to the week of measurement. Between 10:59 AM and 11:10 AM of that day, we observe 1.028 Twitter messages per second (TPS) mentioning Adobe, a level that exceeds the 99^th^ percentile threshold for that firm. Thus, this ten-minute period represents a Twitter spike event that we define as an instance of the treatment. As this time period falls on a Thursday morning, our procedure selects randomly among all other observations for Adobe that occur on some other Thursday morning between 11 and 11:30 am. In this case, the selected control instance was January 17, 2013 beginning at 11:02 am; where the observed Twitter mentions per second (TPS) is below Adobe’s 99^th^ percentile TPS threshold, for the training-window period that ended in the week prior (Jan. 11, 2013)

**Table 1:** Example of a treatment-control group in our data sample.

Symbol	Treatment-group ID	Treatment	Start	End	Tweets /second (TPS)	TPS 80^th^ pctile	TPS 90^th^ pctile	TPS 99^th^ pctile
ADBE	18	0	1/17/13 11:02 AM	1/17/13 11:13 AM	0.175	0.183	0.243	0.878
ADBE	18	1	2/14/13 10:59 AM	2/14/13 11:10 AM	1.028	0.185	0.248	0.802

## Case Studies in the Data

To provide insight into the measurable effects contained in the data, we consider some case studies that we extracted from the data early in the study period in June 2012. These examples, among many others, provide some guidance in operationalizing constructs related to Twitter activity and financial markets reactions at the appropriate levels of granularity in time.

### Akamai: June 5, 2012

**Fig 1:**
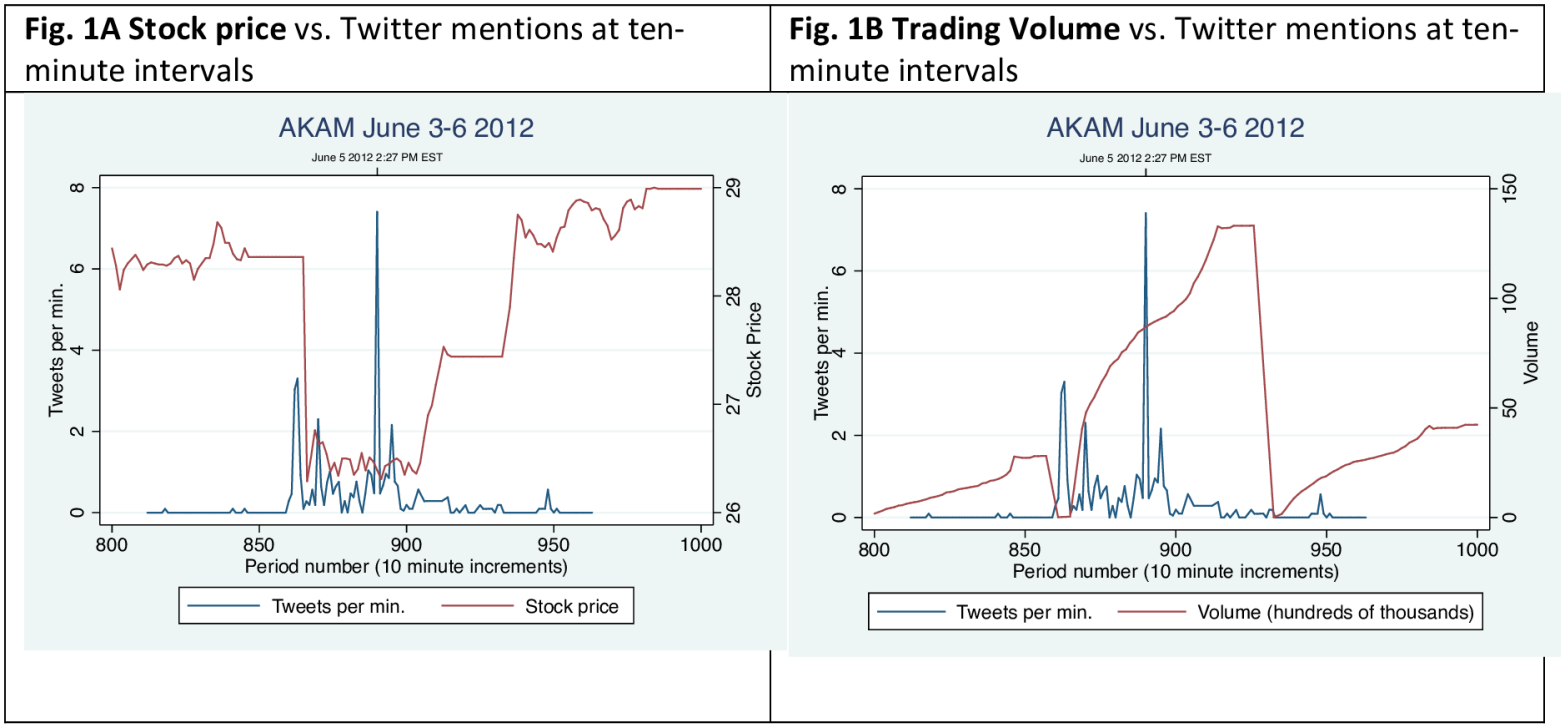
Twitter mentions, stock price, and trading volume for *Akamai*, June 3–6, 2012.

On June 4, 2012, NetFlix announced that it would develop its own content delivery network (CDN) that it called “Open Connect” (openconnect.netflix.com). The news had a sudden impact upon the price and trading volume of stock for Akamai Technologies (AKAM). Akamai is a leading provider of content delivery network services to NetFlix [11]. The prospect of NetFlix becoming no longer reliant on Akamai’s services triggered a burst of trading activity, momentarily bringing down the price of the stock by more than 10%. Within hours, the stock price recovered entirely and the volume of trading resumed to its normal daily cyclical pattern. Several features of the associated Twitter and trading spikes are noteworthy. The spikes of Twitter activity (in blue) are relatively sharp in that they occur over a compressed time frame, as seen in Fig 1. The first spike in Twitter activity precedes the change in stock price or trading volume by approximately ten minutes. A larger spike appears within the hour, after which another flurry of trading activity takes place that restores Akamai to about its original trading price prior to the NetFlix announcement. In comparison to Twitter spikes, the reaction of the financial markets is more gradual and appears to have required a larger amount of time to process and react to new information. Since the reaction of the stock market is transitory, it can be missed entirely at the aggregation level of daily financial returns.

### Garmin: June 11, 2012

**Fig 2:**
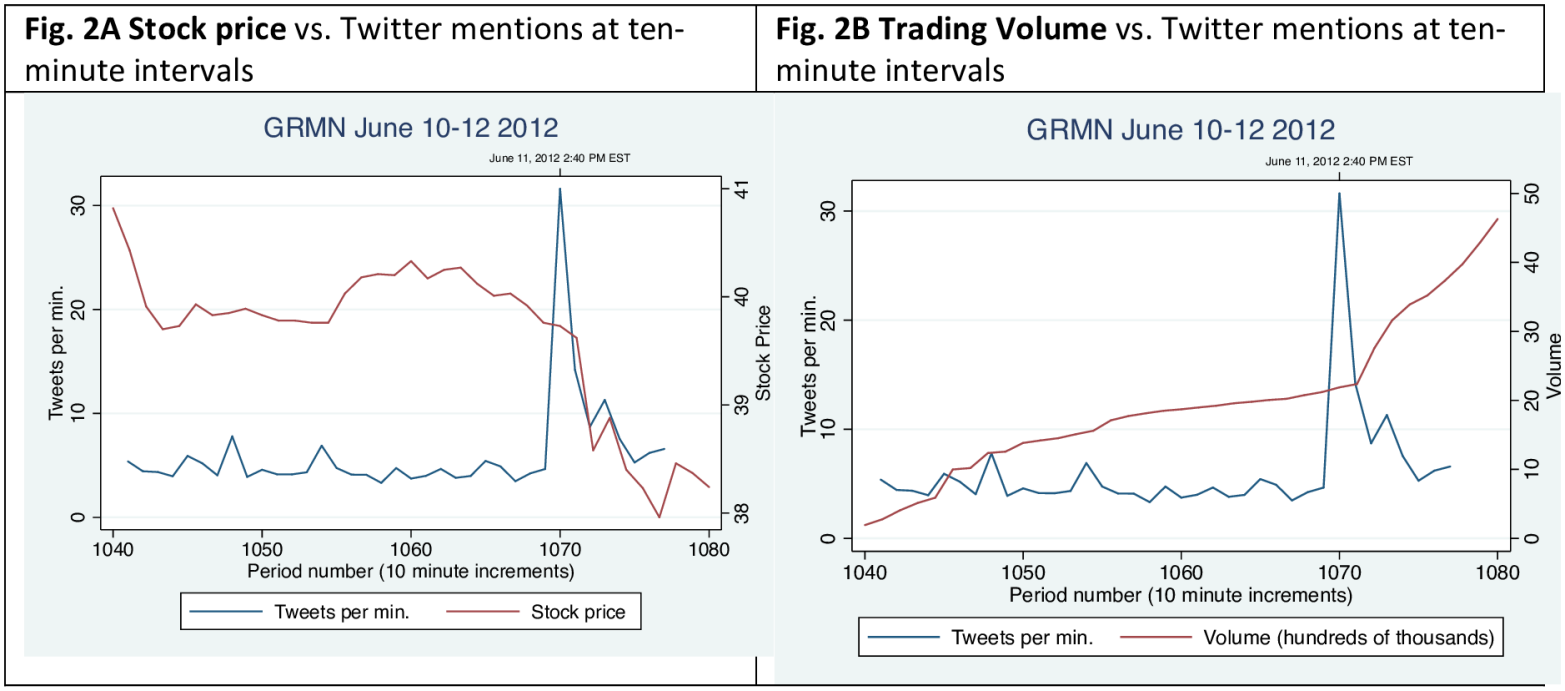
Twitter mentions, stock price, and trading volume for *Garmin*, June 10–12, 2012.

On June 11, 2012, at approximately 2:40 pm Eastern time, Apple announced the launch of new mapping software for its iO6 devices, leading to speculation that its next versions of the iPhone would be equipped with its own voice-enabled GPS service with turn-by-turn navigation [12]. Within minutes, this had a direct impact on Garmin’s stock price and trading volume. Just as in the Akamai case, it is worthwhile to note the difference in the duration and timing between the spike in Twitter mentions and the spikes representing stock price and trading volume. The spike in Twitter activity mentioning Garmin occurs shortly after the announcement and is compressed in a relatively small duration of time, as seen in Fig 2. The frequency of Twitter mentions for this stock quickly returns to normal levels. The reaction in the financial markets takes approximately ten minutes, and it takes at least an hour for the reaction to this news to fully register in both trading volume and price of that stock.

## Empirical Model

The two case examples highlighted above are among many in the dataset that we studied to gain a better understanding of the effects that are present in the data. Exploration of the data suggests that spikes of Twitter activity are much more pronounced over smaller durations of time, whereas the financial market reactions take longer to register the effects and result in more gradual slopes.

### Modeling the Occurrence of a Spike

**Fig 3:**
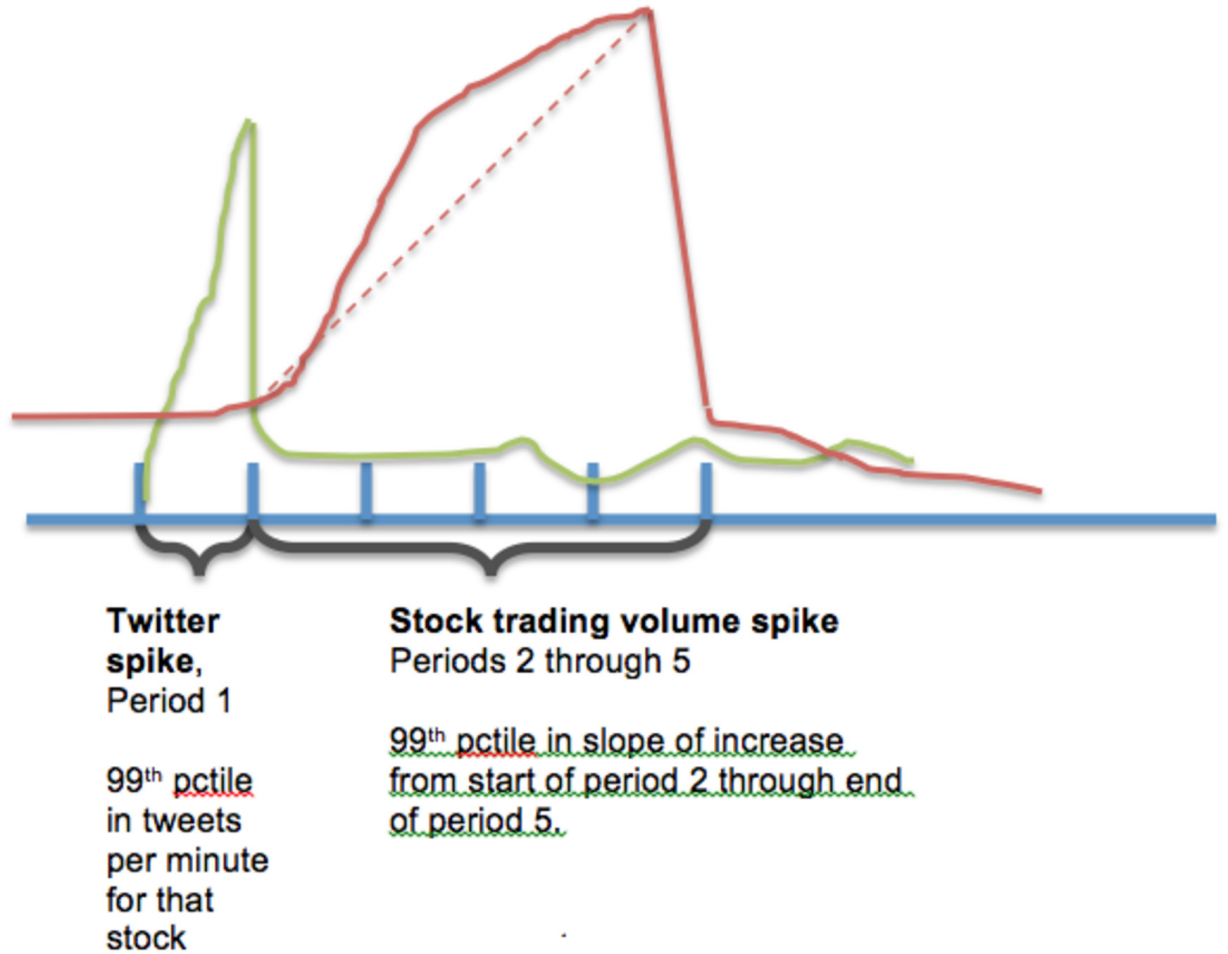
Measurement of spikes in Twitter activity and trading volume for a firm. Each period represents a 10-minute increment of time. The straight dashed-line represents the change in trading volume (which we denote as *ΔTradingVolume40min*) in the 40-minute period subsequent to the period in which the Twitter spike event is observed.

Guided by visual exploration of the data, we define a spike in Twitter activity for a firm as the 99^th^ percentile in the mentions of the common name of the firm per minute, which is calculated with reference to an expanding training-period window that ends prior to the week of measurement. While events exceeded the 99^th^ percentile threshold are probably most salient for identifying spikes, our analysis is broadly consistent when applied at 80^th^ and 90^th^ percentile thresholds. We consider stock market reactions in the forty minutes immediately after a spike in Twitter activity. To capture trading volume spikes, we identify instances in which trading volume reaches the 99^th^ percentile level, as defined based on prior activity for each firm. We illustrate these definitions in Fig 3.

**Fig 4:**
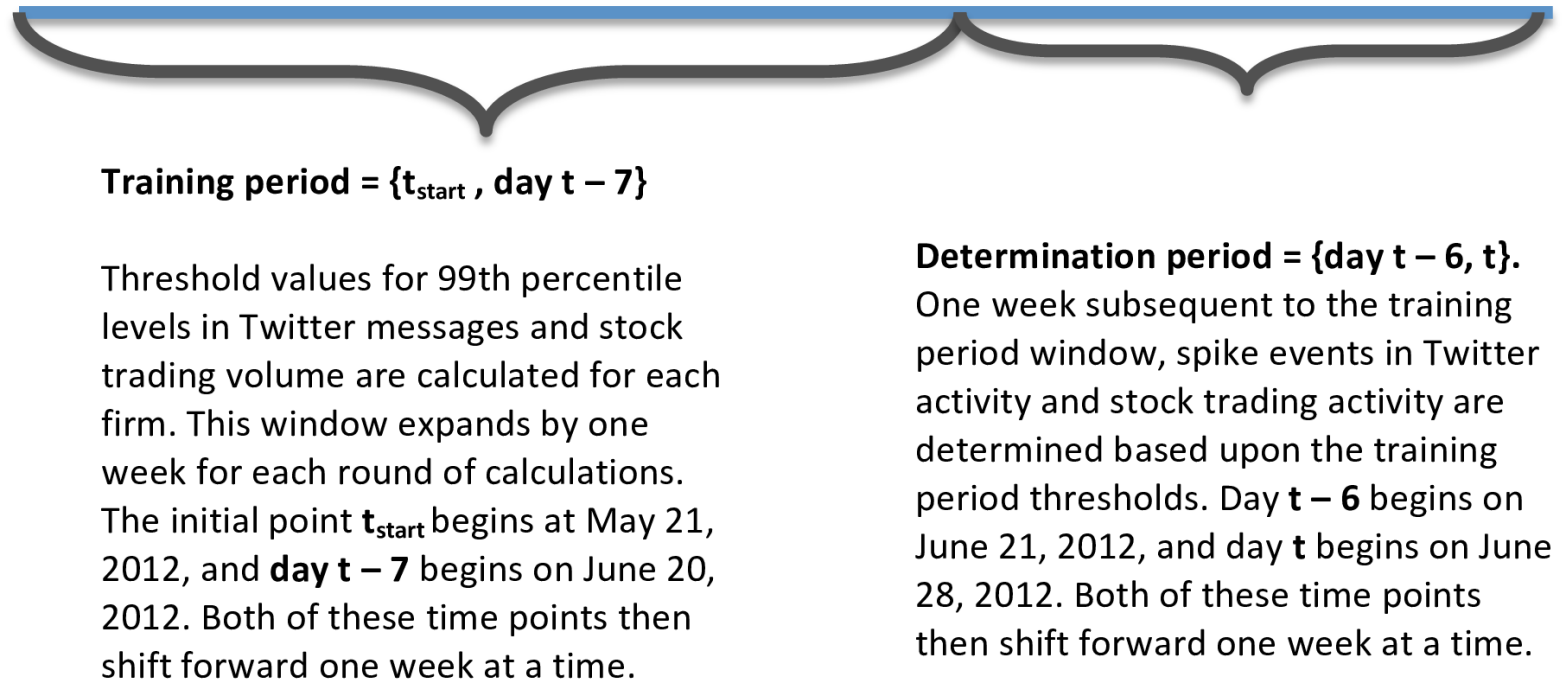
Training and determination periods for 99 percentile spike events.

**Fig 5:**
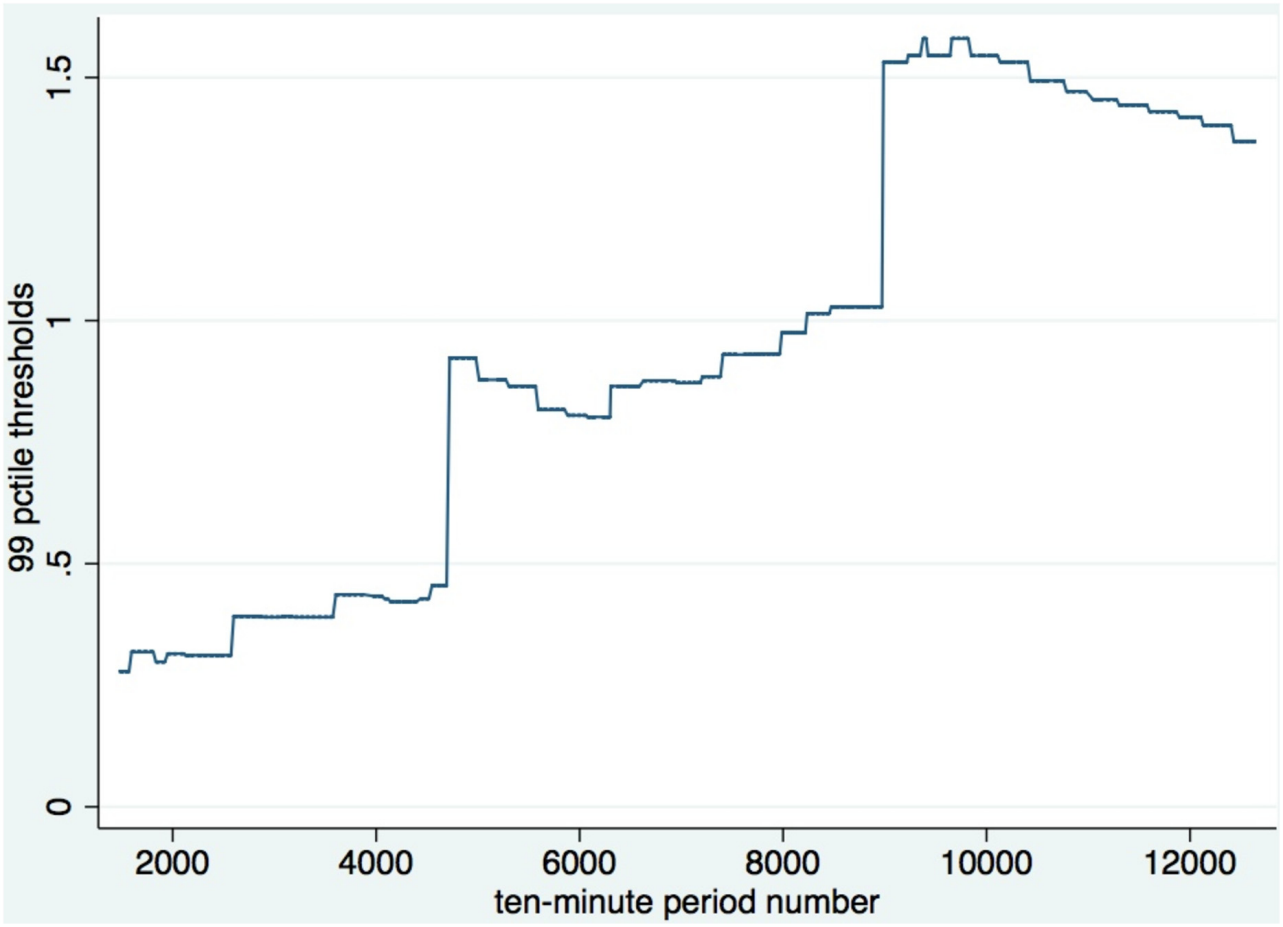
Adobe’s 99^th^ percentile thresholds, from June 21, 2012 through September 17, 2013. The thresholds are updated each week based on all collected past history of data beginning in May 21, 2012. These thresholds are then used to identify spikes for the following week.

To identify spikes in Twitter activity or changes in trading volume, we used a procedure to determine the percentile thresholds beginning with an initial training period comprised of the first month of data. These thresholds are firm-specific; that is, our procedure determines a unique threshold level for each firm that changes each week of the study period based on the trading history of data for that firm ending in the prior week. The training window then expands one week at a time, to determine the percentile threshold levels for each subsequent week. This method ensures that we use only past data to determine the threshold values for spikes in Twitter as well as future financial trading activity. In Fig 4, we illustrate the timeline for the training period and determination period for spike events. The training period expands one week at a time, to identify spikes in the determination period. In Fig 5, we show how Adobe’s 99^th^ percentile thresholds changed each week from June 21, 2012 to September 17, 2013. As shown, the threshold definitions for Adobe are sensitive to two particularly large spikes that occurred in the middle of the study period.

### Changes in trading volume

We define changes in trading volume over the subsequent forty-minutes, which we define in four distinct units of ten-minute increments as in Fig 3. Thus, the subsequent change in trading volume is represented by the following formula:

$$\Delta TradingVolume40min_{i,t}\;=\;TradingVolume_{i,t+4}-TradingVolume_{i,t}$$ (1)

Similarly, we defined the quantities *ΔTradingVolume30min*_*i*,*t*_, *ΔTradingVolume20min*_*i*,*t*_, and *ΔTradingVolume10min*_*i*,*t*_ as changes in trading volume over the subsequent three, two, and single 10-minute increments respectively.

Our general estimation model is a within-estimator (i.e. fixed-effects) to implement a differences-in-differences analysis, in which groups are defined through random treatment-control assignment. To create treatment-control groups, we identified Twitter spike events as treatments and randomly assigned each such observation to a control observation in which Twitter activity was observed to be under the 99^th^ percentile for the firm; consider, for example, how this pairing was done for the Twitter spike listed in Table 1.

Control observations were drawn from past data, and the method for determining the 99^th^ percentile thresholds for eligible controls is based on the expanding training-period window that ends in the week prior to each observation. Random control selections were done conditional on matching of the firm symbol, as well as multiple repeating time indicators such as day of week, hour of day, and half hour. Our four-step procedure for identification of treatment-control groups is outlined in Table 2. This procedure resulted in identification of 5,480 Twitter spike events randomly assigned (with replacement) to control-group observations sharing time of day and day of week indicators at the resolution of a half-hour. In this procedure, a Twitter spike event defines a treatment event. We use a fixed-effects panel model to implement a differences-in-differences approach; fixed-effects are incorporated at the level of the firm and all of the stated time variable indicator units.

**Table 2:** Procedure for Identifying Twitter Spike Events for a Firm.

Step	Description
1) Definition of training and determination period windows	Initial training period window is May 21, 2012 through June 20, 2012. This training window is used to determine Twitter, and stock trading volume spikes at 80^th^, 85th, 90th and 99th percentiles in the initial determination period from June 21, 2012 through June 27, 2012. The training window subsequently expands one week at a time, for identification of spike events in the determination period in the following week; thus the training period window for the subsequent week of June 28, 2012 through July 3, 2012 expands to May 21, 2012 through June 27, 2012; and so on. (see Fig 4)
2) Definition of Twitter spike events	If the number of Twitter mentions in any ten-minute increment exceeds the 99^th^ percentile threshold for the firm in the current week of the study period, based on the training period that ends in the previous week, it is recorded as a Twitter spike event.
3) Control group pool specification	For each firm, we identified a set of historical ten-minute increments during trading hours in which Twitter mentions did not exceed the 99^th^ percentile threshold for that firm, based on the expanding training period window ending in the prior week.
4) Random assignment of treatment to control groups	We matched all of the ten-minute increments in which a Twitter spike event occurred to a ten-minute increment from the control group pool, randomly selecting from matches on the symbol, day of week, hour of day, and half-hour indicators.

Treatment and control groups are defined at ten-minute increments during trading hours between June 21, 2012 and September 18, 2013.

We used a lagged-model framework, such that the levels of Twitter activity are measured for the ten-minute increments immediately preceding the periods in which the dependent variable measures begin:

$$\begin{array}{l} \Delta TradingVolume40min_{i.t}=\\ \;Constant+\;\beta_{1}TwitterSpike_{i,t-1}+\\ \;\beta_{2}\left(NasdaqAvgStockPrice_{i,t-1}-\;NasdaqAvgStockPrice_{i,t-2}\right)/NasdaqAvgStockPrice_{i,t-2}\;+\\ \;\beta_{3}\left(NasdaqAvgVolume_{i,t-1}-\;NasdaqAvgVolume_{i,t-2}\right)/NasdaqAvgVolume_{i,t-2}+u_{i}+\varepsilon_{i,t} \end{array}$$ (2)

We begin with this specific model because overall changes in stock market activity are considered to be strongly associated with the activity for each specific firm. The finance literature has explored time-series patterns in stock market volumes and trading activity in depth; and thus, we do not attempt to replicate that line of scholarship here. We are motivated primarily by how Twitter activity may portend oncoming changes in trading volume. Note that in Eq (2), the subscript *i* represents a treatment-control group, and *t* represents a ten-minute interval. The model in Eq (2) enables us to interpret the estimate of the coefficient β_1_ as the effect of a Twitter spike event in any ten-minute time increment on trading volume in the periods of 40, 30, 20, and 10 minutes beginning in the subsequent ten-minute time increment. The effect is stated as a difference over what may otherwise be expected in the matched control time periods. In other words, the model measures the extent that a Twitter spike for a firm signals an oncoming surge in trading volume, over and above the level that is normally expected for the firm in the given day of week and time of day. We also control for movements in the stock price and trading volume in the prior ten-minute period, averaged over all Nasdaq 100 firms included in the sample.

While we do not posit any kind of causal relationship between chatter on Twitter and movements in the stock market, our model allows us to examine whether chatter on Twitter pertaining to a firm can serve as a signal to predict unusually high levels of trading volume in subsequent minutes within the next hour. Because trading activity for a given firm can be unusually high in regularly occurring intervals, for example in a time of the day for a particular day of the week (for example, Tuesdays between 10 and 10:30 am), our model accounts for this through the treatment-control group matching.

We also code the dependent variable as a binary indicator representing unusually high increases in trading volume or changes in stock market price, at the 99^th^ percentile based on an expanding training-period window. To identify trading volume events, we used the same expanding training-period window that was used to identify spikes in Twitter activity, again to ensure that only past data was used to define the thresholds that mark these events. We used firm-specific thresholds at the 99^th^ percentile to define unusual upward surges in trading volume. Here again we use a within-estimation empirical framework, this time implementing a differences-in-differences model through a logistic fixed-effects panel estimator appropriate for binary dependent variables. Eq (3) expresses this model:

$$\begin{array}{l} log{\left(odds\;of\;trading\;volume\;surge\right)}_{i.t}=\;Constant+\;\beta_{1}TwitterSpike_{i,t-1}+\\ \;\beta_{2}\left(NasdaqAvgStockPrice_{i,t-1}-\;NasdaqAvgStockPrice_{i,t-2}\right)/NasdaqAvgStockPrice_{i,t-2}\;+\\ \;\beta_{3}\left(NasdaqAvgVolume_{i,t-1}-\;NasdaqAvgVolume_{i,t-2}\right)/NasdaqAvgVolume_{i,t-2}+u_{i}+\varepsilon_{i,t} \end{array}$$ (3)

Dependent variable definitions are presented in Table 3, and control variables are presented in Table 4.

**Table 3:** Dependent Variables.

Variable Name	Variable Construction/ Definition	Data Source
ΔTradingVolume [40min, 30 min, 20min, 10min]_i,t_	Change in total number of shares traded of the stock from the beginning of the subsequent ten minute time increment, measured over periods of 10, 20, 30, and 40 minutes.	Yahoo!Finance
Trading Volume Event: 99th pctile	Binary variable indicating an excess of the 99^th^ percentile in ΔTradingVolume; based on expanding training window ending in the prior week. A value of one means that a spike in trading volume occurs in that ten-minute period.	Yahoo!Finance

**Table 4:** Control Variables: Definitions and Data Sources.

Variable Name	Variable Construction/ Definition	Data Source
Nasd100 avg. trading volume change (t-1)	Prior period percentage change in average Nasdaq 100 trading volume: [(NasdaqAvgVolume_t-1_-NasdaqAvgVolume_t-2_)/ NasdaqAvgVolume_t-2_].	Yahoo!Finance
Nasd100 avg. stock price (t-1)	Prior period percentage change in average Nasdaq 100 stock price: [(NasdaqAvgStockPrice_t -1_- NasdaqAvgStockPrice_t -2_)/ NasdaqAvgStockPrice_t -2_].	Yahoo!Finance
Fixed-effects units in treatment-control groups	Firm, day of week, hour of day, and half-hour	

## Main Findings

Table 5 shows the results of paired t-tests comparing the magnitude in changes in trading volume within each treatment-control group pairing. The differences between Twitter spike events and their matched control group counterparts are statistically significant (at p < 0.0001). The paired t-test results suggest that a Twitter event is associated with an increase in trading volume of about 562,141 shares, on average. This is significant in magnitude, as the median in trading volume during the sample periods is 1,191,554 shares; thus the average effect represents about 47% of the median in total shares traded. We took steps to address the concern that statistical significance is not an artifact of the large sample size, by random selection of sub-samples of 250 treatment-control group pairs; see e.g. [13]. We conducted the same t-tests several dozen times using different sub-samples each time, and one example is shown in the bottom row of Table 5. We found that the t-test statistics for trading volume remained strongly significant in every sub-sample instance. Our random sub-sample results suggest that the average treatment effects (ATE) for trading volume are robust and insensitive to sample size.

**Table 5:** Comparison of changes in trading volume following Twitter Spike Events and Non-Event Control periods: Paired t-tests.

	Twitter Spike Event (Treatment)		No Twitter Spike (Control)		Difference	Comparison test
	Mean (m_t)	Std. Err.	Mean (m_c)	Std. Err.	m_t—m_c (std. err.)	Paired t-test of Ha: |m_t—m_c| > 0
**Main Sample: 5,480 treatment-group pairs**						
*ΔTradingVolume*_*40min*_	1,067,836	47,648	505,694	13,849	562,141 (45,152)	t = 12.4; p < 0.0001
**Randomly selected sub-sample: 250 treatment-group pairs**						
*ΔTradingVolume*_*40min*_	826,132	95,769	522,726	70,889	303,406 (95,120)	t = 3.19; p < 0.0001

We next consider the results of the fixed-effects panel implementations of the difference-in-differences, which we present in Tables 6 and 7. Because the panel units in the fixed-effects models are the same treatment-control groups based upon indicators of firm, day of week, and half-hour of day, they are basically the same as the paired t-tests, except that they incorporate additional controls for one-period lagged movements in overall average Nasdaq trading volume and stock price. These control variables are important because overall movements in the stock market can influence the trading of any particular stock. The results in Tables 6 through 8 also show side-by-side comparisons of the different effects for 40, 30, 20 and 10 minutes. Trading volume increases are greater following a Twitter spike event than they would otherwise be: About 116,000 shares greater over the course of ten minutes, to about 509,000 shares over forty minutes, as indicated in Table 6 results. As expected, these magnitudes are in line with the results of the paired t-test. Just as we did with the paired t-tests, we conducted the same panel regressions on a number of randomly selected sub-samples of 250 treatment-group pairs. The observed Twitter spike effects on subsequent trading volume are invariably statistically significant at p < 0.01 in the dozens of randomly selected sub-samples that we have tested. One example of the small sub-sample results for trading volume is reported in Table 7. We find results consistent to those reported in Table 7 among many randomly selected sub-samples, alleviating concerns that the observed effect upon trading volume may be an artifact of the central limit theorem for large samples [13].

**Table 6:** Effect of Twitter Event on Subsequent Trading Volume: Differences-in-differences with treatment-control group fixed-effects.

	(1)	(2)	(3)	(4)
Subsequent time period:	40 minutes	30 minutes	20 minutes	10 minutes
Twitter event (t-1)	509,018***	392,807***	262,407***	116,204***
	(41,999)	(34,390)	(23,800)	(8,850)
Nasd100 avg. stock price chg (t -1)	3.679e+08***	2.982e+08***	1.693e+08***	1.073e+08***
	(5.832e+07)	(4.776e+07)	(3.305e+07)	(1.229e+07)
Nasd100 avg. trading volume chg (t -1)	-1,291	-912.5	-814.5	-638.5
	(2,921)	(2,392)	(1,655)	(615.6)
Observations	10,960	10,960	10,960	10,960
Treatment-control groups	5,480	5,480	5,480	5,480
F stat	63.11***	57.31***	49.93***	84.29***

Fixed-effects panel regressions within treatment-control groups, created by matching ten-minute time increments randomly by firm, day of week, hour of day, and half-hour units.

Standard errors in parentheses. Significant at *10%, **5%, and ***1% level for two-tailed t-tests.

**Table 7:** Small-sample Effect of Twitter Event on Subsequent Trading Volume: Differences-in-differences with treatment-control group fixed-effects.

	(1)	(2)	(3)	(4)
Subsequent time period:	40 minutes	30 minutes	20 minutes	10 minutes
Twitter event (t-1)	340,376***	216,821***	125,694***	51,526**
	(98,738)	(71,855)	(37,436)	(20,389)
Nasd100 avg. stock price chg (t -1)	2.913e+08	1.940e+08	1.281e+08*	4.151e+07
	(1.794e+08)	(1.305e+08)	(6.801e+07)	(3.704e+07)
Nasd100 avg. trading volume chg (t -1)	-4,512	-1,433	-4,416	-3,560*
	(9,843)	(7,163)	(3,732)	(2,032)
Observations	500	500	500	500
Treatment-control groups	250	250	250	250
F stat	4.9***	3.9**	5.3***	3.3**

Fixed-effects panel regressions within treatment-control groups, created by matching ten-minute time increments randomly by firm, day of week, hour of day, and half-hour units. Standard errors in parentheses. Significant at *10%, **5%, and ***1% level for two-tailed t-tests.

**Table 8:** Effect of Twitter Event on Likelihood of Subsequent Spikes in Trading Volume: Logistic panel fixed-effects on main sample.

	Main Sample Trading Volume Event: 99th pctile
Twitter event (t-1)	2.219***
	(0.157)
Nasd100 avg. stock price	209.9
change (t-1)	(214.0)
Nasd100 avg. trading volume change (t-1)	0.00230
	(0.00778)
Observations	942
Treatment-control groups	471
Chi-sqr stat	357.7***

Differences-in-differences through logistic panel fixed-effects model within control groups assigned randomly by the firm, day of week, hour of day, and half-hour units. Final subsample includes only identified treatment-control groups that feature at least one trading volume event and one non-event; where the event is defined as a 99^th^ percentile Trading Volume change, based upon prior trading activity of the firm. Thus, this is a smaller sample than in Table 6. Standard errors in parentheses. Significant at *10%, **5%, and ***1% level for two-tailed t-tests.

Table 8 shows the fixed-effects panel logistic regression estimation results for the model in Eq (3), where the dependent variable is defined as a binary indicator representing an unusual surge in subsequent trading volume over forty minutes; using the same spike-identification method detailed in Table 2. Sample size is smaller in this model, because it requires both a zero and one outcome (in trading-volume spike events) in each treatment-control group. According to the estimation results in Table 8, periods featuring a spike in Twitter mentions of a firm have a greater odds of being followed by an upsurge in trading volume by a factor of exp(2.219), representing approximately a 9-fold increase in the odds ratio.

## Predictive Power of Tweets

In order to compare the explanatory analyses from the previous section to the predictive power of Tweets, we conducted forecasting exercises to predict trading volume changes in the subsequent forty minutes for each time period during the trading day. For time period t representing a ten-minute interval, the models predict the change in trading volume from periods t + 1 to t + 4. Forecasting was done with OLS regression models that were trained upon expanding one-week windows. We built an initial model on a training period that began on July 1, 2012 and ended on March 31, 2013, and that expanded one-week at a time to form predictions in each subsequent week. This model is specified below in Eq (4).

The forecasting model includes an indicator variable for each firm to account for firm fixed-effects; and separate binary indicators representing Twitter spike events at the 80^th^, 90^th^, and 99^th^ percentiles. These Twitter spikes are identified using the methods detailed in Table 2, ensuring that the definition of these spikes is based only on events that would have occurred prior to each time period. We also include levels of Twitter activity as well as the square, cube and logarithm of these Twitter messaging levels. The model also includes the prior forty-minute change in trading volume; for any ten-minute interval t, this is the change in trading volume from period t– 5 through period t– 1. We include the square and the log of this term. We also included binary indicators for day of week (*DayOfWeek*), hour of day (*HourOfDay*), and half hour (*FirstHalfHour*, coded as 1 if time period t is within the first thirty minutes of the hour, 0 otherwise).

$$\begin{array}{l} \Delta TradingVolume40min_{\left(t+1\;to\;t+5\right),\;i}=\\ \;\beta_{1}TwitterSpike99pctile_{i,t-1}+\;\beta_{2}TwitterSpike90pctile_{i,t-1}+\\ \;\beta_{3}TwitterSpike80pctile_{i,t-1}+\;\beta_{4}TweetsPerSecond_{i,t-1}+\;\beta_{5}TweetsPerSecond_{i,t-1}{}^{2}+\;\\ \;\beta_{6}TweetsPerSecond_{i,t-1}{}^{3}+\;\beta_{7}\text{ln}\left(TweetsPerSecond_{i,t-1}+1\right)+\\ \;\beta_{8}\Delta TradingVolume40min_{\left(t-5\;to\;t-1\right),\;i}+\;\beta_{9}{\left(\Delta TradingVolume40min_{\left(t-5\;to\;t-1\right),\;i}\right)}^{2}+\\ \;\beta_{10}\text{ln}\left(\Delta TradingVolume40min_{\left(t-5\;to\;t-1\right),\;i}+1\right)+\sum \beta_{d}{\text{DayOfWeek}}_{\text{d}}\;+\\ \;\sum \beta_{\text{h}}{\text{HourOfDay}}_{\text{h}}+FirstHalfHour+u_{i}+\;\varepsilon_{i,t} \end{array}$$ (4)

In the above, the individual firm fixed effect is modeled as u_i_. We also run forecasting models that omit Twitter-related predictors represented by coefficients β_1_ through β_7_, but include all other predictors listed in Eq (4). Inclusion of the seven Twitter-related predictors has no effect on the adjusted R^2^ in regression estimates of Eq 4. Values of R^2^ and adjusted R^2^ are the same, both at approximately 0.71, whether or not the seven terms are included in the model. This is probably because of the large number of regressors already in the model: Note that the fixed effect u_i_ is short-hand notation referring to 95 additional coefficient terms in the regression model, one for each firm in the sample except a reference unit, in addition to indicator variables for day of week and hour/half-hour of day. With one observation for each ten-minute period for each firm, the sample size of the first training window period ending on March 31, 2013 is 321,796 data points, and subsequently grows each week as the training window expands. We use the resulting training model coefficient estimates to produce running forecasts in the subsequent week.

**Fig 6:**
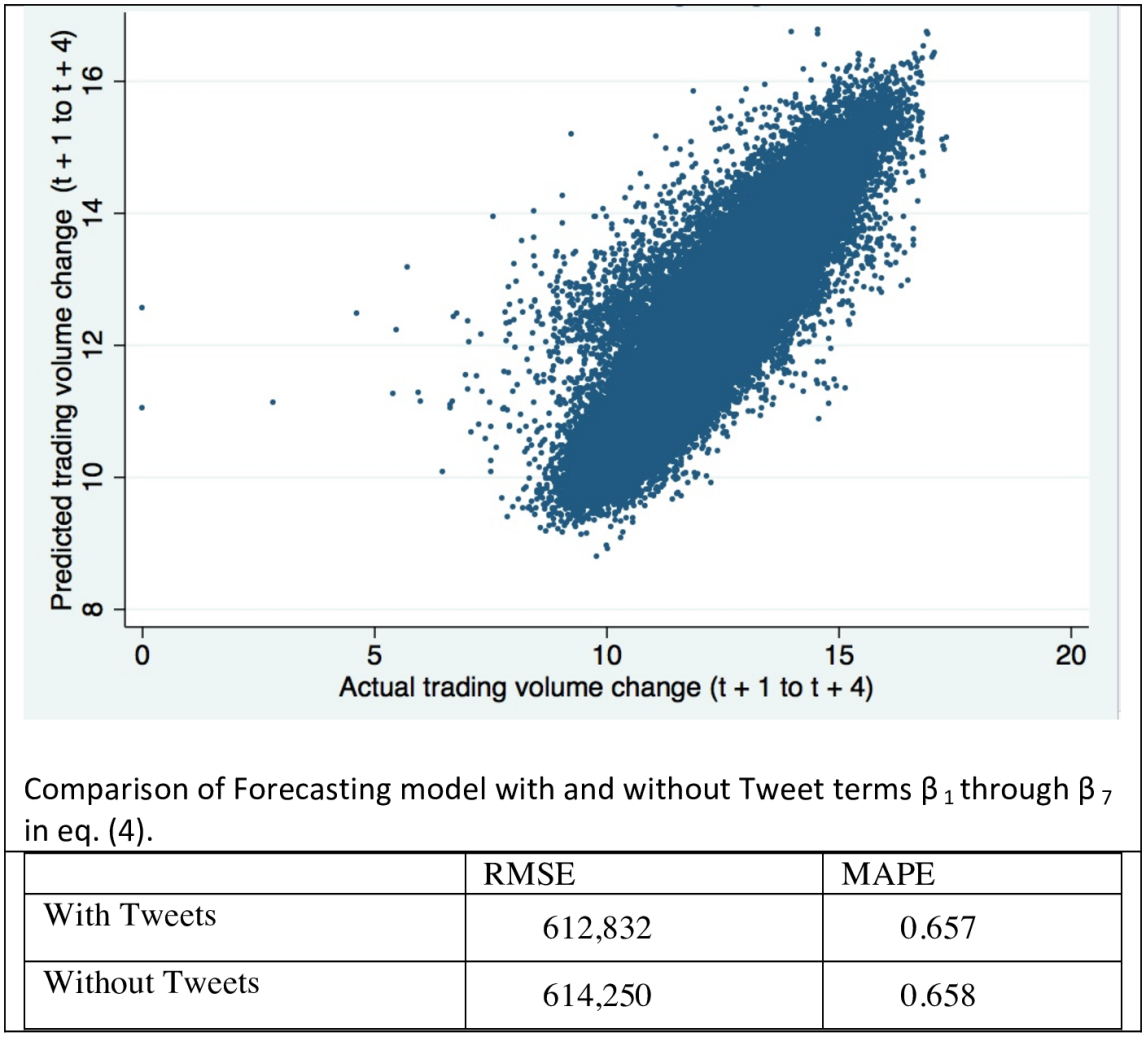
Actual vs. Predicted trading volume change, Log-Log scale: 134,072 observations comprising ten-minute periods during trading hours in the forecasting period between April 1, 2013 and June 7, 2013.

The results are shown in Fig 6. The plot of actual versus predicted values of ΔTradingVolume40min_i,(t+1 to t + 5)_ show a rather well-defined scatter around the diagonal line. This suggests that there is no systematic bias in our predictions. The values of RMSE and MAPE suggest that inclusion of the Twitter-related predictors into our forecasting model results in slightly improved predictions. The level of improvement, in the order of magnitude of 0.2%, does not appear very large. However, day traders and high frequency traders might find even a 0.2% advantage per trade quite lucrative. Yet, our results may also suggest that in order to forecast more accurately, one may have to steer away from interpretable linear models to more black-box algorithmic models; see also [10]. We did attempt to use more flexible functional forms for forecasting, by expanding Eq (4) with non-linear interaction terms and higher-order powers of the terms, and rather than seeing improvements in the forecasts, we started to see indications of over-fitting that made the predictions less accurate. Thus, while we find a compelling and potentially informative relationship between Twitter activity and trading volume in real-time, our forecasting exercise highlights how difficult it can be to make use of this information for monetary gain. Perhaps more can be done by exploiting the sentiment in the content of the Twitter messages, combined with more sophisticated algorithmic models (but which may also be more of a black box and allow for less insight than the models that we are employing). This is an opportunity for future research.

## Discussion

In practice, it is extremely difficult for individual investors to capitalize on newly released public information by trading in the stock markets. The speed of information exchange, ever-faster financial trading networks, and liquidity of financial markets, all ensure that market prices almost instantly absorb news as it is released to the public, denying arbitrage opportunities to all but exclusive groups of institutional traders [14]. For all practical purposes as far as common investors are concerned, the efficient market hypothesis is robust in denying arbitrage opportunities in the stock market based upon newly released public information [15].

Nevertheless, news can spread on Twitter much more quickly than it can be absorbed in the financial markets. For an individual person, the act of sending or relaying a message on Twitter is often fast and effortless, without immediate financial consequences. Executing a trade on the stock market based on the same information can be a cognitively and emotionally taxing process that requires more time. Thus, information may spread on large social networks such as Twitter before the financial markets can process it.

Our results suggest that a spike in chatter on large-scale social networks such as Twitter about any firm may signal an impending surge in trading activity of the firm’s stock, whether or not the Twitter spike is causal. This not only presents an opportunity to harness valuable information for participants in financial markets; but also provides greater insight into the types of information that spread on large-scale social networks such as Twitter. We observe the potential for distinct speeds at which information diffuses in Twitter in comparison to the time it takes for financial traders to process and act upon that information. We observe effects that are statistically significant, transitory, and require finite amounts of time. As such, signals propagating in Twitter may be useful to traders seeking to exploit small delays in the diffusion of news and the relatively slow responsiveness of the markets. We believe that statistical models employing real-time data from large social networks can apply not just to the financial markets, but also to other areas of electronic commerce in which consumer sentiment can have measurable effects in real-time, such as in online auctions or online merchandising. Moreover, from the perspective of industry research, it is possible to reveal and quantify more clearly the dependencies between firms in an industry ecosystem.

## Supporting Information

S1 Dataset Data sets used in the final analysis. A compressed.zip file containing two comma delimited files. The file named TwitterYahooFinalSample_PLoSOne.csv contains data used for Tables 5–8. The file named TwitterYahooPredictRangeDec2015_forPLoSOne.csv contains data for Fig 6.
(ZIP)

S1 List of Firms An Excel file (.xlsx) containing the stock tickers of the 96 firms tracked in this study and the common names used to identify them within the Twitter messages. (XLSX)

S1 Mapping of Variable Names to Data Fields A document that maps the key variables in the paper to the corresponding field names in the dataset. (DOCX)
