# Supplementary material for: Real-Time Diffusion of Information on Twitter and the Financial Markets
Source: PLoS One. 2016 Aug 9;11(8):e0159226. doi: 10.1371/journal.pone.0159226 (PMC4978482; doi:10.1371/journal.pone.0159226)
Supplement: S1 Mapping of Variable Names to Data Fields — (DOCX) [file pone.0159226.s003.docx]

**Mapping of Variable Names to Data Fields**

Key variables and corresponding fields in the dataset provided for ***Real-time Diffusion of Information on Twitter and the Financial Markets*,** by Ali Tafti, Ryan Zotti and Wolfgang Jank.

| **Variable name** | **Table or equation in paper** | **Field name in the dataset** |
| --- | --- | --- |
| *ΔTradingVolume40min_i,t_* | Table 3 | df4_vol_f1 |
| *ΔTradingVolume30min_i,t_* | Table 3 | df3_vol_f1 |
| *ΔTradingVolume20min_i,t_* | Table 3 | df2_vol_f1 |
| *ΔTradingVolume10min_i,t_* | Table 3 | df1_vol_f1 |
| *Trading Volume Event:*  *99th pctile* | Table 3 | isev4vol_99pct_f1 |
| *TwitterSpike_i,t-1_* | Eq. 2, Eq. 3 | istreatment |
| *Nasd100 avg. stock price chg (t -1 ) = (NasdaqAvgStockPrice_t-1_ – NasdaqAvgStockPrice_t-2_)/NasdaqAvgStockPrice_t-2_* | Table 4 | abs_nasd100smpl_stch_l1 |
| *Nasd100 avg. trading volume chg (t -1 ) = (NasdaqAvgVolume_t-1_ – NasdaqAvgVolume_t-2_)/NasdaqAvgVolume_t-2_* | Table 4 | nasd100smpl_volch_l1 |
| *Firm identifier* | Table 4 | symbol |
| *Day of week identifiers (Mon-Friday)* | Table 4 | dow1-dow5 |
| *Hour of day, half-hour* | Table 4 | hod5-hod9, first_halfhour |

| **Additional data fields of importance** | **Description** |
| --- | --- |
| eligible | Boolean value indicating that the data point is used in testing of equations 2 and 3, because it is within a control-treatment group pairing. |
| treatgroup_id | Treatment-group identifier used as the fixed-effects unit for testing of equations 2 and 3. |
